# Production of the Growth Factors GM-CSF, G-CSF, and VEGF by Human Peripheral Blood Cells Induced with Metal Complexes of Human Serum ***γ***-Globulin Formed with Copper or Zinc Ions

**DOI:** 10.1155/2014/518265

**Published:** 2014-07-03

**Authors:** Sergey B. Cheknev, Maria A. Apresova, Nadezhda A. Moryakova, Irina E. Efremova, Anna S. Mezdrokhina, Lidya S. Piskovskaya, Alla A. Babajanz

**Affiliations:** Laboratory of Cell to Cell Interactions, N.F. Gamaleya Research Institute of Epidemiology and Microbiology, Health Ministry of the Russian Federation, Gamaleya Street 18, Moscow 123098, Russia

## Abstract

As it was established in our previous studies, the proteins of human serum *γ*-globulin fraction could interact with copper or zinc ions distributed in the periglobular space, form metal complexes, and become able to perform effector functions differing due to the conformational shifts from those mediated by them in native conformation of their Fc regions. In the present work we have evaluated ability of the *γ*-globulin metal complexes formed with copper or zinc ions in the conditions like to the physiological ones to induce production or to regulate induction in the culture of freshly isolated human peripheral blood cells (PBC) of granulocyte (G) and granulocyte-macrophage (GM) colony-stimulating factors (CSF) as well as of vascular endothelial growth factor (VEGF). The *γ*-globulin metal complexes formed with both copper and zinc ions were found to similarly reduce production of GM-CSF, G-CSF, and VEGF induced in normal human PBC cultures by the control *γ*-globulins or by copper and zinc ions used alone. In context of theory and practice of inflammation the properties of the *γ*-globulin metal complexes might impact the basic knowledge in search of novel approaches to anti-inflammatory drugs development.

## 1. Introduction

Inflammatory process, as known, involves recruitment of different cell types that produce cytokines and chemokines at part initiating migration of reactive cells to the lesions of inflammation or activating the cells attracted to the damaged tissues [1–5].

Between cytokines that play key roles in initiation and development of inflammation interferon-*γ* (IFN-*γ*), interleukin-1*β* (IL-1*β*), IL-2, IL-6, IL-12, and tumor necrosis factor *α* (TNF-*α*) are produced by activated T cells or macrophages and monocytes (MC), by dendritic cells (DC) and other cell types beginning from the first steps of inflammation till the termination phase of the process [1–5]. They act as factors of DC1/DC2, MC1/MC2, and (or) Th1/Th2 polarization of the inflammatory response and form inflammatory environment for cells functioning at the tissue lesions [1, 2, 6–8]. They activate many types of responsive cells, amplify cell to cell interactions, and force the functions of neutrophils, lymphocytes, MC, and macrophages as well as DC migrating and operating in the inflammatory loci [1, 2, 9].

On the other hand, some kinds of growth factors appear to be involved in the inflammatory process because, in the contrary to “classic” cytokines, they especially induce and accelerate migration of the responsive cells, attraction of them to the tissue lesions, and obtaining ability to migrate and to exhibit regulatory or inflammatory properties in the loci of tissue damage [5, 8, 10–13]. GM-CSF, G-CSF, and also VEGF serve as chemoattractants directly involved in inflammatory process. They are considered to play a primordial role of proinflammatory cytokines produced in physiological state but strongly accumulated in the damaged tissues [8, 14–18]. They recruit neutrophils, lymphocytes, or MC into the inflammatory lesions, where the cells introduce in regulatory network supporting productive phase of inflammatory response [13, 19–21].

The proteins of human serum *γ*-globulin fraction that include the full pool of circulating natural antibodies do present a family of factors able to induce inflammation due to their binding with Fc fragment receptors (FcR) followed by stimulation of intracellular signal transduction pathways that leads to the induction of proinflammatory cytokine production by the responsive cells and to involvement of the cells expressing activatory FcR in the inflammatory inducing cytokine network [22–25]. Fc*γ*RIII bearing cells enhance expression of the genes of IFN-*γ* and TNF-*α* after activation with the IgG Fc portion [26–28], also they augment production of IFN-*γ*, TNF-*α*, as well as, IL-1*β*, and IL-6 in this state of activation [22, 29]. Therefore, they not only induce and enhance inflammation but also shift inflammatory response to the Th1 type [22, 25, 26].

As was shown in our studies of the past ten years, the proteins of human serum *γ*-globulin fraction exerted ability to chelate copper or zinc ions from the periglobular space and to pass because of such interactions through structural transformations with primary localization of them at the Fc region of the protein molecules [30, 31]. Transformed with metal ions chelating *γ*-globulins have not shown any new antigen specificity but were found to obtain new effector properties compared with those performed by *γ*-globulins in native Fc region conformation [30, 32]. According to the metal specificity of the complexes formed the latest ones demonstrated reciprocal or similar effects on the cytokine production by responsive cells, and in a comparison with the action of control *γ*-globulins were found to induce or inhibit production of IFN-*γ*, IL-1*β*, IL-2, and IL-6 by normal human PBC [33–35], to similarly enhance production of TNF-*α*, IL-18, and IL-10 [36–38], or to have no effects with reference to IL-4 or IL-17 [38]. They could contribute thereby to the initiation and supporting inflammatory process, and their effects seem to significantly differentiate them from those of *γ*-globulins in native Fc region conformation [33–37].

Also, being activated due to IgG ligation, Fc*γ*R bearing cells enhance expression of the genes of GM-CSF and (or) VEGF and accumulate GM-CSF in the pool of synthetized cytokines [27, 39]. On the other hand, production of GM-CSF has been shown to be induced with IL-1*β*, IL-6, and TNF-*α* [8] and enhanced in presence of IL-18 [5]. Production of VEGF mRNA was found to be induced by IL-1*β* and IL-6 [40, 41] and enhanced in presence of IL-1*β*, IL-6, or TNF-*α* [20].

It was not surprising to suggest that human serum *γ*-globulins due to chelating the copper or zinc ions from the periglobular space, and because of conformational shifts caused by such protein to metal interactions could change dynamics of their binding to Fc*γ*R and intracellular signal transduction responsible for the induction or inhibition of GM-CSF, G-CSF, and VEGF production. Actually, as it has been shown in this work, the *γ*-globulin metal complexes formed with both copper and zinc ions in the conditions like to the physiological ones were found to similarly reduce production of GM-CSF, G-CSF, and VEGF induced in normal human PBC cultures by the control *γ*-globulins or by copper and zinc ions used alone.

## 2. Materials and Methods

### 2.1. Preparation of the Samples of Human Serum *γ*-Globulin Metal Complexes Formed with Copper or Zinc Ions

For preparation of the human *γ*-globulin metal complexes, the human serum *γ*-globulin (ICN) dissolved at the dose of 100.0 *μ*g/mL in 0.15 M NaCl (in tablets of Eco-Service) solution and supplemented with 1.0 M or 0.1 M NaOH (Chemapol) up to pH 6.84–7.06 was firstly cleared from big associates by the membrane filtration through pores of the diameter of 0.45 *μ*m (Millipore). Then the samples of *γ*-globulin were incubated with adding copper sulphate CuSO_4_ × 5H_2_O (Merc) or zinc chloride ZnCl_2_ (Baum-Lux) containing 0.5 *μ*g/mL of copper or zinc ions, respectively, in 0.15 M NaCl solution or with adding 0.15 M NaCl solution alone in the volume corresponding to that containing copper or zinc salts (control proteins). The incubation was carried out in the total volume of 10.0 mL of the sample in 15 mL polypropylene centrifuge tubes (Corning) for 1 hr at 37°C for both experimental (with metal ions) and control (without metal ions) *γ*-globulins.

Following incubation, to separate an excess of the metal ions which were not bound by the proteins at the previous step of preparation, the samples underwent two steps Ultracel-30 K (Amicon) molecular ultrafiltration at 1700 g, 5 min, slow cooling, in the volume of 10.0 mL of each one with intersteps resolving in the initial volume (10.0 mL) of 0.15 M NaCl solution.

The samples were restored with accuracy in the volume of 5.0 mL of 0.15 M NaCl solution, which were then taken out of the tubes, and were analyzed spectrophotometrically with ultraviolet (UV) ranged between 190 and 320 nm of the wave length with the interval of testing of 0.1 nm using differential spectrophotometer UV-1800 Shimadzu. Also the UV analysis of the experimental and control samples was performed before and after the incubation of the *γ*-globulins with metal ions or without them.

Following sterilization of the samples obtained by passing them through the protein suitable membranes with the pores of the diameter of 0.45 *μ*m (Millipore) they were frozen and stored at −18°C till the time of investigation on the cell cultures.

### 2.2. Determination of the Quantities of Copper or Zinc Ions Bound per One Protein Molecule

The concentration of copper collected in the filtrate of Ultracel-30 K (Amicon) two steps molecular ultrafiltration was detected with use of the complex forming reaction of copper with 10^-3 ^M sodium diethyldithiocarbamate trihydrate (Aldrich) in 0.15 M NaCl solution supplemented with 0.1 M NaOH (Chemapol) up to pH 9.0–9.2. Photometric registration of the reaction was applied at the wave length of 440 nm (UV-1800 Shimadzu).

The concentration of zinc collected in the filtrate as described above was detected with use of the complex forming reaction of zinc with o-phenanthroline (Bio N) ranged by the doses used between 3.0 × 10^−5^ and 7.0 × 10^−5^ M in 0.15 M NaCl solution in neutral range of pH. Photometric registration of the reaction was applied at the wave length of 225 nm (UV-1800 Shimadzu).

After the count of quantities of copper or zinc ions collected in the filtrates the determination of quantities of metal ions bound per one protein molecule was performed based on real *γ*-globulin concentration measured through the photometric estimation of the optical density at the wave length of 280 nm and the recount using the extinction coefficient of 0.7. The results obtained show that the *γ*-globulin metal complexes prepared in the work and then used for the study on the cell cultures contained 5 and 8 copper ions or 12 and 18 zinc ions bound per one molecule of the *γ*-globulin.

The state of pH at the full consequence of the samples preparation was controlled with use of the basic pH-meter Sartorius PB-11 supplied with PY-P11 electrode.

### 2.3. Obtaining and Preparation of PBC Cultures

The cultures of freshly isolated normal human PBC were prepared from the probes of heparinized (5.0 U/mL of heparine, Moscow Endocrine Factory) peripheral venous blood of healthy subjects obtained from the Moscow Blood Transfusion Station. The probes of the whole blood were diluted with the RPMI-1640 medium (Pan Eco) supplemented with 2.0 v/v % of the donor's plasma, L-glutamine (accessory to the medium), and 20.0 U/mL of gentamycin (Biochemist), till the quantity of the PBC became 10^6^ cells per 1 mL of suspension.

### 2.4. Induction of Cytokines

Induction of the cytokine pool containing GM-CSF, G-CSF, and VEGF was carried out on PBC suspensions obtained and prepared as described above and passed into the wells of 24 well flat bottom plastic plates (Costar) in the volume of 2.0 mL of the suspension into each well. PBC were incubated for 24, 48, and 72 hrs at 37°C in humid atmosphere containing 5% CO_2_.

The samples of human serum *γ*-globulin metal complexes formed with copper or zinc ions were added to the PBC suspensions for obtaining the final concentration of the proteins that was equal to 0.5 *μ*g/mL, once after the suspensions passing, and were not removed from the wells throughout the cytokine induction. Also, in parallel with estimation of the effects of the metal *γ*-globulin complexes the action of control *γ*-globulin samples as well as of copper (II) sulphate and zinc chloride was tested. The salts were prepared just to contain the same quantities of copper or zinc ions as those bound per one protein molecule in the metal *γ*-globulin complexes and expressed through their concentrations. Control *γ*-globulins used for the cytokine induction were utilized after fully undergoing the course of the *γ*-globulin metal complexes preparation but did not contain metal ions. Also, as the *γ*-globulin metal complexes, they contained 0.5 *μ*g/mL of the protein and were initially supplemented with the 0.15 M NaCl solution in the corresponding volumes.

Newcastle disease virus (NDV) of the vaccine H strain (Deposit SCV2348, L.A. Tarasevich State Institute for Standardization and Control of Medical Biological Preparations) at the concentration of 10 cytopathic doses per one cell and phytohemagglutinin P (PHA, Pan Eco) at the dose of 1.0 *μ*g/mL were used as the standard cytokine inducers.

Each step of the induction (24, 48, or 72 hrs of the cells incubation) was followed by the accurate collection of the cell supernates into the 15 mL polypropylene centrifuge tubes (Corning), which were cooled and stored at 4°C. When the final probes (72 hrs of the cells incubation) were collected, the samples of PBC culture medium were dispensed in the volume of 500 *μ*L into the 1.5 mL Eppendorf tubes for each one and then were frozen and stored at −18°C till the time of the cytokine testing.

### 2.5. Measurement of the GM-CSF, G-CSF, and VEGF in the PBC Supernates

The concentrations of GM-CSF, G-CSF, and VEGF induced on the normal human PBC cultures were determined using immunoenzyme analysis and ELISA Processor II (Behring). The systems of the Vector Best Europe Company were applied accordingly to the instructions of the manufacturer, with additional technological control probes.

Each sample of each supernate obtained was tested at the primary state and at its dilution 1/10 with RPMI-1640 medium (Gibco). Each dilution of each the sample of supernates and of the controls used (NDV, PHA, and *γ*-globulins which underwent the initial membrane filtration only, and spontaneous cytokine production by PBC) were tested not less than in two parallel wells of microplates.

### 2.6. Statistical Analysis

The mean values and the mean's errors were calculated. The Student's *t*-test was used for determination of the statistical significance of differences between the mean values. The count was made with use of the Microsoft Office Excel mathematic program.

## 3. Results and Discussion

### 3.1. Production of GM-CSF by Normal Human PBC Induced with Human Serum *γ*-Globulin Metal Complexes Formed with Copper or Zinc Ions

The data obtained indicate that GM-CSF did appear in the culture medium of normal human PBC induced with *γ*-globulin metal complexes, as well as with their control proteins or metal ions used alone not earlier than after 48 hrs of the cells incubation.

Only PHA and NDV induced GM-CSF production by normal human PBC during the first 24 hrs of the cells incubation. The level of production corresponded to 38.38 ± 0.63 pg/mL and to 188.5 ± 5.37 pg/mL respectively. Spontaneously PBC did produce 4.38 ± 0.75 pg/mL of GM-CSF that was approximately 1.73–3.18 times more than in presence of *γ*-globulin metal complexes and their protein or metal controls (*P* < 0.02–0.1). The control proteins did induce production of 1.85 ± 0.21 pg/mL and 2.08 ± 0.21 pg/mL of GM-CSF, zinc ions used alone—of  2.53 ± 0.13 pg/mL, copper ions used alone—of  2.48 ± 0.11 pg/mL, *γ*-globulin complex with zinc ions—of  2.45  ±  0.10 pg/mL, and *γ*-globulin complex with copper ions—of 1.38 ± 0.18 pg/mL of GM-CSF (data not shown).

**Figure 1:**
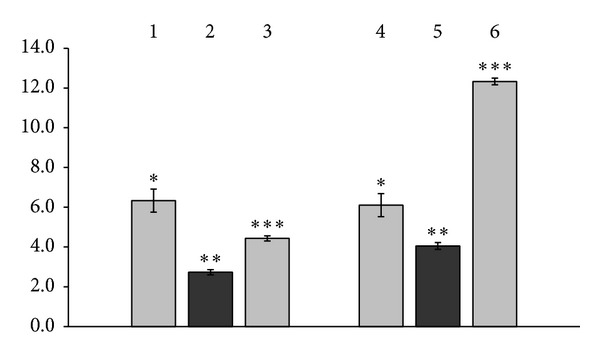
Production of GM-CSF by normal human PBC induced with human serum *γ*-globulin metal complexes formed with copper or zinc ions, with control *γ*-globulins, and with copper and zinc ions used alone (*M* ± *m*, *n* = 4). Induction for 48 hrs at 37°C. In the ordinate axis: concentration of GM-CSF, pg/ml. Here and at Figures 2 and 3: 1—control *γ*-globulin for the zinc complex, 2—*γ*-globulin complex formed with zinc ions, 3—zinc ions used alone, 4—control *γ*-globulin for the copper complex, 5—*γ*-globulin complex formed with copper ions, and 6—copper ions used alone. Here and at the Figures 2 and 3: the concentrations were calculated from the data obtained with using primary (not diluted) state of the samples. *P < 0.05 compared with zinc ions used alone (3) or with *γ*-globulin complex formed with copper ions (5); **P < 0.01 compared with control *γ*-globulin for the zinc complex (1) or with *γ*-globulin complex formed with zinc ions (2); ***P < 0.001 compared with *γ*-globulin complex formed with zinc ions (2) or with zinc ions used alone (3), with control *γ*-globulin for the copper complex (4) and with *γ*-globulin complex formed with copper ions (5).

As seen in Figure 1, the second 24 hrs of the cells incubation (48 hrs of GM-CSF induction) led to the production of more significant amounts of GM-CSF. The control proteins did induce production of 6.1 ± 0.58 pg/mL and 6.33 ± 0.58 pg/mL of GM-CSF, zinc ions used alone—of  4.43 ± 0.13 pg/mL, copper ions used alone—of  12.33 ± 0.17 pg/mL whereas *γ*-globulin complex with zinc ions did induce production of 2.73 ± 0.13 pg/mL, and *γ*-globulin complex with copper ions—of  4.05 ± 0.17 pg/mL of GM-CSF (Figure 1). Spontaneous GM-CSF production in normal human PBC cultures at 48 hrs of observation corresponded to 2.75 ± 0.13 pg/mL, PHA induced production by normal human PBC of 178.63 ± 8.88 pg/mL of GM-CSF, and NDV—of  55.0 ± 3.49 pg/mL of GM-CSF (data not shown).

It seems to be evident that due to metal chelation human serum *γ*-globulin became slower in the ability to induce GM-CSF production by normal human PBC compared with the control proteins and metal ions used alone. The complex with zinc ions exerted 2.32 times reduced GM-CSF inducing activity than the control protein (*P* < 0.01, Figure 1) and 1.62 times reduced induction of GM-CSF production by normal human PBC than zinc ions themselves (*P* < 0.001, Figure 1). The complex with copper ions exhibited 1.51 times reduced GM-CSF inducing properties than the control *γ*-globulin (*P* < 0.05, Figure 1) and 3.04 times reduced induction of GM-CSF production by normal human PBC than copper ions themselves (*P* < 0.001, Figure 1).

Later, during the next 24 hrs of the cells incubation (until 72 hrs of GM-CSF induction) the levels of GM-CSF production by normal human PBC in presence of *γ*-globulin metal complexes as well as of control proteins and metal ions used alone returned to those of spontaneously producing cells. They did not overcome spontaneous 2.78 ± 0.22 pg/mL of GM-CSF and did not differ significantly from spontaneous PBC production. The mentioned above levels of GM-CSF production ranged between 2.42 ± 0.09 pg/mL and 2.99 ± 0.12 pg/mL. PHA induced production by normal human PBC of 335.5 ± 27.19 pg/mL and NDV—of  15.88 ± 0.28 pg/mL of GM-CSF (data not shown).

### 3.2. Production of G-CSF by Normal Human PBC Induced with Human Serum *γ*-Globulin Metal Complexes Formed with Copper or Zinc Ions

The data obtained indicate that unlike the effects of GM-CSF induction G-CSF was produced by normal human PBC as cytokine of the early response.

Not only PHA (41.38 ± 1.02 pg/mL of G-CSF induced) or NDV (1.09 ± 0.04 ng/mL of G-CSF induced) but also control human serum *γ*-globulins served as G-CSF inducers in which presence normal human PBC were able to produce up to 100.0 pg/mL of G-CSF, although such a production was found to vary significantly. The complex with zinc ions did induce production by normal human PBC of not more than 9.66 ± 0.41 pg/mL of G-CSF likely to zinc ions themselves in which presence the cells were able to produce not more than 6.33 ± 0.85 pg/mL of G-CSF (data not shown). The complex with copper ions did induce production by normal human PBC of 21.38 ± 0.65 pg/mL of G-CSF similarly with copper ions used alone in presence of which the cells produced 17.33 ± 0.62 pg/mL of G-CSF (data not shown). Spontaneous G-CSF production by normal human PBC was determined as 9.39 ± 0.54 pg/mL (data not shown).

**Figure 2:**
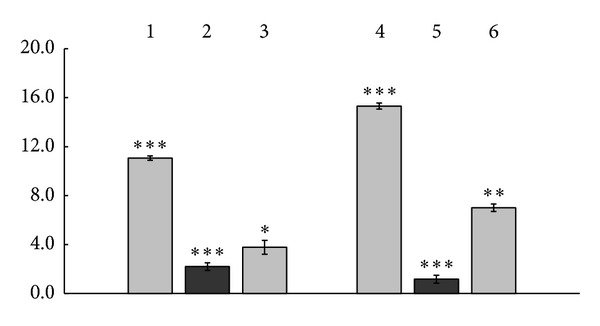
Production of G-CSF by normal human PBC induced with human serum *γ*-globulin metal complexes formed with copper or zinc ions, with control *γ*-globulins, and with copper and zinc ions used alone (*M* ± *m*, *n* = 8). Induction for 48 hrs at 37°C. In the ordinate axis: concentration of G-CSF, pg/ml. *P < 0.1 compared with *γ*-globulin complex formed with zinc ions (2); **P < 0.01 compared with zinc ions used alone (3); ***P < 0.001 compared with zinc ions used alone (3) or with control *γ*-globulin for the zinc complex (1), as well as compared with copper ions used alone (6) or with control *γ*-globulin for the copper complex (4) and with copper ions used alone (6).

The second 24 hrs of the cells incubation (48 hrs of G-CSF induction) led to reducing response of normal human PBC to activation with control *γ*-globulins that was accompanied by augmentation of G-CSF concentrations found in presence of zinc and copper ions themselves with reference to spontaneous G-CSF production by normal human PBC. As seen in the Figure 2 the control proteins did induce production of 11.06  ±  0.18 pg/mL and 15.31 ± 0.25 pg/mL of G-CSF, zinc ions used alone—of  3.78 ± 0.56 pg/mL, copper ions used alone—of  7.01 ± 0.30 pg/mL whereas *γ*-globulin complex with zinc ions did induce production of 2.2 ± 0.31 pg/mL, and *γ*-globulin complex with copper ions—of  1.17 ± 0.32 pg/mL of G-CSF (Figure 2) that was even 2.67 times lower than the level of G-CSF produced spontaneously by normal human PBC (*P* < 0.05). Spontaneous G-CSF production by normal human PBC cultures at 48 hrs of observation corresponded to 3.12 ± 0.49 pg/mL, in presence of PHA the cells produced 35.94 ± 0.70 pg/mL of G-CSF, in presence of NDV 3.65 ± 0.32 ng/mL of G-CSF could be detected in the culture medium (data not shown).

Being summarized the data obtained at 48 hrs of PBC incubation indicate that because of metal chelation the human serum *γ*-globulin also, as with reference to GM-CSF production, lost ability to induce G-CSF production by normal human PBC compared with the control proteins and metal ions used alone. The complex with zinc ions exerted 5.03 times reduced G-CSF inducing activity than the control protein (*P* < 0.001, Figure 2) and 1.72 times reduced induction of G-CSF production by normal human PBC than zinc ions themselves (*P* < 0.1, Figure 2). The complex with copper ions exhibited 13.1 times reduced G-CSF inducing properties than the control *γ*-globulin (*P* < 0.001, Figure 2) and 6.0 times reduced induction of G-CSF production by normal human PBC than copper ions themselves (*P* < 0.001, Figure 2).

During the next 24 hrs of the cells incubation (until 72 hrs of G-CSF induction) production of G-CSF almost completely abolished in normal human PBC cultures. The levels of G-CSF production were not detected in presence of control *γ*-globulins, of their complexes with zinc ions or of zinc ions used alone, or corresponded to spontaneous G-CSF production by normal human PBC. The latest one was found as 0.73 ± 0.10 pg/mL of G-CSF. The protein complex with copper ions did induce production of 2.0 times greater amounts of G-CSF (1.45 ± 0.17 pg/mL, *P* < 0.05) and copper ions used alone did induce production of 1.83 times higher G-CSF concentrations than PBC did spontaneously (1.33 ± 0.30 pg/mL, *P* > 0.1) (data not shown). In presence of PHA the cells produced 40.75 ± 1.44 pg/mL of G-CSF, in presence of NDV 4.14 ± 0.05 ng/mL of G-CSF could be detected in the culture medium (data not shown).

### 3.3. Production of VEGF by Normal Human PBC Induced with Human Serum *γ*-Globulin Metal Complexes Formed with Copper or Zinc Ions

The data obtained indicate that VEGF did appear in the culture medium of freshly isolated normal human PBC at the first 24 hrs of the cells incubation. It was produced spontaneously at the level of 167.19 ± 3.11 pg/mL with following augmentation by the presence in the cell supernates up to 341.25 ± 6.66 pg/mL (*P* < 0.001) at 48 hrs of the cells incubation and later up to 597.19 ± 13.54 pg/mL (*P* < 0.001) at 72 hrs of VEGF induction (data not shown).

During the first 24 hrs of observation as well as at the time of 72 hrs of the cells incubation VEGF production by normal human PBC in presence of *γ*-globulin metal complexes or of their protein and ionic controls also, like to augmentation in culture of unstimulated normal human PBC, increased but not exceeded spontaneous activity of the cells (data not shown). Only at the time of 48 hrs of the cells incubation VEGF inducing potency of *γ*-globulins and of metal ions used alone could be registered.

At 24 and 72 hrs of the study the control proteins induced production of VEGF ranged between 90.94 ± 2.79 pg/mL and 94.38 ± 2.98 pg/mL and between 515.0 ± 14.06 pg/mL and 544.38 ± 13.63 pg/mL, respectively; zinc ions used alone did induce production of 32.19 ± 1.67 pg/mL and 566.88 ± 12.69 pg/mL of VEGF, copper ions used alone—of  65.31 ± 1.20 pg/mL and 521.25 ± 13.77 pg/mL, respectively, the complex with zinc ions—of 48.44 ± 0.94 pg/mL and 511.25 ± 16.46 pg/mL, and the complex with copper ions—of 49.38 ± 1.32 pg/mL and 511.88 ± 30.84 pg/mL of VEGF, respectively (data not shown). Production of VEGF by normal human PBC induced in presence of PHA corresponded to 48.13 ± 0.63 pg/mL and 265.94 ± 3.78 pg/mL and in presence of NDV—to  76.25 ± 2.95 pg/mL and 310.0 ± 8.18 pg/mL, respectively.

**Figure 3:**
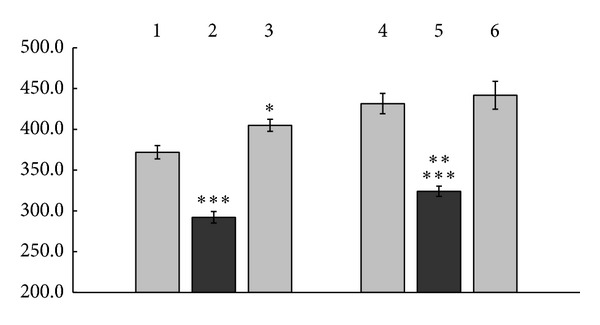
Production of VEGF by normal human PBC induced with human serum *γ*-globulin metal complexes formed with copper or zinc ions, with control *γ*-globulins, and with copper and zinc ions used alone (*M* ± *m*, *n* = 8). Induction for 48 hrs at 37°C. In the ordinate axis: concentration of VEGF, pg/ml. The absciss axis is conducted from the concentration of 200.0 pg/ml. *P < 0.1 compared with control *γ*-globulin for the zinc complex (1); **P < 0.05 compared with *γ*-globulin complex formed with zinc ions (2); ***P < 0.001 compared with control *γ*-globulin for the zinc complex (1) and with zinc ions used alone (3), as well as being compared with control *γ*-globulin for the copper complex (4) and with copper ions used alone (6).

As Figure 3 demonstrates, the control proteins did induce at 48 hrs of the cells incubation production of 371.88 ± 8.22 pg/mL and 431.56 ± 12.45 pg/mL of VEGF, zinc ions used alone—of  405.0 ± 7.40 pg/mL, copper ions used alone—of 441.88 ± 17.12 pg/mL whereas *γ*-globulin complex with zinc ions did induce production of 292.19 ± 7.05 pg/mL, and *γ*-globulin complex with copper ions—of 324.06 ± 6.16 pg/mL of VEGF (Figure 3).

As seen in Figure 3, the complex of both *γ*-globulin formed with zinc ions and *γ*-globulin transformed with copper ions chelation showed decrease in induction of VEGF production by normal human PBC compared with not only their protein or ionic controls but also spontaneous VEGF production (the latest data not shown). The complex with zinc ions exerted 1.27 times reduced VEGF inducing activity than the control protein (*P* < 0.001, Figure 3) and 1.39 times reduced induction of VEGF production by normal human PBC than zinc ions themselves (*P* < 0.001, Figure 3). The complex with copper ions exhibited 1.33 times reduced VEGF inducing properties than the control *γ*-globulin (*P* < 0.001, Figure 3) and 1.36 times reduced induction of VEGF production by normal human PBC than copper ions themselves (*P* < 0.001, Figure 3).

PHA induced at 48 hrs of the cells incubation production by normal human PBC of 112.19 ± 4.83 pg/mL of VEGF. In presence of NDV production of 205.31 ± 6.10 pg/mL of VEGF could be determined (data not shown).

## 4. Discussion

As it was established in our previous studies, human serum *γ*-globulins exerted ability to chelate copper or zinc ions from the periglobular space, and to pass because of such interactions through structural transformations with primary localization of them at the Fc region of the protein molecules [30, 31]. Structural basis for these interactions and transformations followed from the primary amino acid sequence of human IgG1 with its secondary, tertiary, and quaternary configurations including availability of special structures of hinge region as well as of oligosaccharide chains expression on the surface of C_H_2 domains. It was determined by our detailed analysis [30, 31] and was recently confirmed by the group of Sibéril et al. [42] also suggesting that metal ions chelation by the proteins of *γ*-globulin fraction, especially by IgG1, might be considered as a reaction closely related to the consequence of physiological events.

As a result of the mentioned above interactions human serum *γ*-globulins obtained the effector functions which differentiated them from those mediated by the proteins in native conformation of their Fc fragments. The complex formed with copper ions served as an efficient inducer of IFN-*γ* and early IL-2 production [33]. The complex formed with zinc ions exhibited induction of the early IL-1*β* [35]. The complexes formed with both copper and zinc ions exerted induction of IL-6 and TNF-*α* production [34, 36].

At the same time, in contrast to the effects of *γ*-globulin complex formed with copper ions the complex originated from the interaction of proteins with zinc ions decreased induction of IFN-*γ* and early IL-2 production by normal human PBC incubated with the control *γ*-globulins [33]. Also the complex with copper reciprocally to the action of the zinc complex one decreased induction of early IL-1*β* by the cells responding to the presence of control *γ*-globulin [35]. Moreover, both *γ*-globulins complexed with copper ions and proteins transformed with zinc ions binding induced production by normal human PBC of IL-10 [38].

Thus, as enhancement of inflammation with forcing Th1 polarization of the immune response and inhibition of inflammatory reactions with their shift towards Th2 differentiation might present an attribute of *γ*-globulin/metal ions interaction, which result would be determined by full the majority of influences generating in the inflammatory and anti-inflammatory network.

As it could be seen from the mentioned above data the balance of proinflammatory and anti-inflammatory signals generated due to *γ*-globulin/metal ions interactions and their consequent effector performance is tightly regulated by the mechanisms originated from own properties of *γ*-globulin metal complexes. It seems to be evident that for each inducing signal its downregulating and limiting effect in the network exists. Actually, IFN-*γ* and early IL-2 production by normal human PBC which increased in presence of the *γ*-globulin complex with copper ions were reduced under influence of *γ*-globulin complex with zinc [33]. Early IL-1*β* production by normal human PBC which increased in presence of the *γ*-globulin complex with zinc ions was reduced under influence of *γ*-globulin complex with copper [35]. Both the complexes of human serum *γ*-globulin formed with copper and zinc ions exerted induction of IL-6 and TNF-*α* production [34, 36] and complexes with both zinc and copper induced production by normal human PBC of IL-10 [38] which, as known, serves as a functional antagonist of TNF-*α* and IL-6 and limits their effects in cytokine regulatory network.

The growth factors studied as well as the family of Fc*γ*R, briefly reviewed in the Introduction, also serve the factors involved in both induction and inhibition of inflammation and balanced intercellular interactions enhancing or decreasing the state of inflammation. G-CSF was found to induce expression by the cells of Fc*γ*RI [11]. GM-CSF was shown to enhance production of TNF-*α* which is potentiated by the action of IFN-*γ* [18]. Simultaneously, Fc*γ*RIII induces IL-10 production [43] that inhibits induction of the synthesis of GM-CSF induced in presence of IL-1, IL-6, and TNF-*α* [8], as well as enhancement of VEGF production by TNF-*α*, IL-1*β*, and IL-6 [20]. Fc*γ*RIIA limits expression of IL-10 and Th2 differentiation [44] whereas Fc*γ*RIIB controls expression of IL-12, production of IFN-*γ*, and differentiation of Th1 [44–46].

It has been indicated in the Introduction that proteins of human serum *γ*-globulin fraction exchanging by the metal ions with other blood plasma macromolecules do participate in the initiation, prolongation, and termination of inflammation due to their ability to bind Fc*γ*R of the responding cells and to induce or inhibit intracellular signal transduction pathways which are involved in the inflammatory reactions [22–29]. A part of activity of the *γ*-globulin fraction proteins is dealing with enhancement of the expression of some growth factors following IgG ligation by the Fc*γ*R bearing cells which are also involved in the inflammatory or anti-inflammatory effects [5, 8, 20, 27, 39–41].

The present study demonstrates that human serum *γ*-globulins exerted induction of the production by normal human PBC of GM-CSF (Figure 1), G-CSF (Figure 2), and VEGF (Figure 3). Independently of them, copper and zinc ions used alone also exhibited the properties of GM-CSF, G-CSF, and VEGF inducers (Figures 1, 2 and 3) with the expression of the inducing effects of copper ions in 1.85–2.79 times (*P* < 0.001–0.01) higher extent compared to the action of zinc (Figures 1 and 2).

Being chelated by the proteins of human serum *γ*-globulin fraction, copper or zinc ions lost essential part of their growth factors inducing potency (Figures 1, 2 and 3). In parallel *γ*-globulins due to structural transformations caused by the metal chelating reduced their own PBC inducing activity (Figures 1, 2 and 3). As a consequence of such transformations the protein complexes formed following *γ*-globulin/metal ions interactions demonstrate significantly reduced potency to induce production by normal human PBC of GM-CSF (Figure 1), G-CSF (Figure 2), and VEGF (Figure 3) compared with the effects of control *γ*-globulins and metal ions used alone. Concerning G-CSF and VEGF induction in presence of *γ*-globulin metal complexes formed with both copper and zinc ions normal human PBC produce growth factors in up to even 1.17–2.67 times less extent than they do spontaneously (*P* < 0.01–0.05, data not shown).

It would be rational to agree with observation indicating on relatively short period of time (around 48 hrs of induction) in which PBC demonstrate decline in growth factors production in response to the action or presence of *γ*-globulin metal complexes. But taking into account that induction of such key cytokines of inflammatory process as IFN-*γ*, IL-2, IL-1*β*, IL-6, TNF-*α*, and inhibitory IL-10, due to the properties of *γ*-globulin metal complexes, might be supported at the balanced state (see above), coincidence in reduced production of GM-CSF, G-CSF, and VEGF by normal human PBC could reflect dynamic shift in the cell activity illustrating achievement by PBC of their functional state beginning which generation of inflammatory inducing signals provided by the growth factors would be reduced.

## 5. Conclusions

The data obtained allow suggesting that metal exchange between blood plasma macromolecules impacts in steady state balance of proinflammatory and anti-inflammatory signals. In context of such a balance ensuring *γ*-globulins bound metal ions generate signals involved in inflammation both induction and termination. When the antigenic or mitogenic forcing appears they would decrease induction of the production of GM-CSF, G-CSF, and VEGF by normal human PBC could temporarily reduce a flow of inflammatory inducing signals provided by the growth factors and might probably be considered as perspective ones in context of further development of new anti-inflammatory therapeutics.

The questions remain about cell populations and the receptor groups involved in mechanisms of regulation by the *γ*-globulin metal complexes of growth factors production, as well as about possibility for practical appliance of the results obtained. They need arrangement of special experiments and further detailed analysis.
